# Increased expression and copy number amplification of LINE-1 and SINE B1 retrotransposable elements in murine mammary carcinoma progression

**DOI:** 10.18632/oncotarget.1188

**Published:** 2013-08-09

**Authors:** Alberto Gualtieri, Federica Andreola, Ilaria Sciamanna, Paola Sinibaldi-Vallebona, Annalucia Serafino, Corrado Spadafora

**Affiliations:** ^1^ Istituto Superiore di Sanità, Rome, Italy; ^2^ Institute of Translational Pharmacology, CNR, Rome, Italy; ^3^ Department of Experimental Medicine and Surgery – University of Rome “Tor Vergata”, Rome, Italy

**Keywords:** LINE-1 retrotransposons, SINE retrotransposons, reverse transcriptase, copy number amplification, breast cancer progression

## Abstract

In higher eukaryotic genomes, Long Interspersed Nuclear Element 1 (LINE-1) retrotransposons and endogenous retroviruses represent large families of repeated elements encoding reverse transcriptase (RT) proteins. Short Interspersed Nuclear Element B1 (SINE B1) retrotrasposons do not encode RT, but use LINE-1-derived RT for their retrotransposition. We previously showed that many cancer types have an abundant endogenous RT activity. Inhibition of that activity, by either RNA interference-dependent silencing of active LINE-1 elements or by RT inhibitory drugs, reduced proliferation and promoted differentiation in cancer cells, indicating that LINE-1-encoded RT is required for tumor progression. Using MMTV-PyVT transgenic mice as a well-defined model of breast cancer progression, we now report that both LINE-1 and SINE B1 retrotransposons are up-regulated at a very early stage of tumorigenesis; LINE-1-encoded RT product and enzymatic activity were detected in tumor tissues as early as stage 1, preceding the widespread appearance of histological alterations and specific cancer markers, and further increased in later progression stages, while neither was present in non-pathological breast tissues. Importantly, both LINE-1 and SINE B1 retrotransposon families undergo copy number amplification during tumor progression. These findings therefore indicate that RT activity is distinctive of breast cancer cells and that, furthermore, LINE-1 and SINE B1 undergo copy number amplification during cancer progression.

## INTRODUCTION

A strikingly unexpected finding emerging after the completion of the human genome sequencing indicates that protein-coding genes make up a mere 1.2% of the human genome, while the rest of the genomic DNA is devoid of protein-coding functions [1]. These data have radically modified the traditional view that only protein-coding genes were at the heart of genome function. The non-coding portion of the genome has been found to be pervasively transcribed, and various classes of non-coding RNAs (ncRNAs) operate in multiple genome-wide regulatory mechanisms (for reviews see [2-3]). These findings have “rehabilitated” the non-coding portion of the genome (long dismissed as functionally irrelevant, or selfish [4-5] ‘junk’ DNA [6]), with unexpected implications of novel genetic mechanisms in tumorigenesis [7-10]. A wealth of studies have actually disclosed global regulatory roles for ncRNAs [11-12], small RNA families [13], ultra conserved regions (UCRs) [14-15] and retrotransposable elements (retroelements, [16-17]). The latter account for about 45% of the human genome [1, 18] and can be subdivided into two large families, i.e. long terminal repeat (LTR)-containing endogenous retroviruses (HERVs) and non-LTR retrotransposons, which include LINE-1 and SINE-VNTR-Alu (SVA) families [18]. HERV and LINE-1 are autonomously replicating elements: the former closely resemble infectious retroviruses in structure, while the latter comprise two open reading frames, ORF1 and ORF2 [18-19]: ORF2 encodes the RT product, which enables them to move within the genome via a “copy-and-paste” mechanism involving the reverse transcription of RNA intermediates and the insertion of the resulting cDNA copies in the host genome. SINEs lack the RT-encoding gene and exploit the retrotransposition machinery provided by LINE-1 elements [17-18].

Retroelements are implicated in a variety of diseases [20] including cancer [21]. Growing data indicate that the expression of retrotransposable elements, and of the RT protein product, is low or absent in differentiated tissues [22-23] and up-regulated in embryonic [24-26] and transformed cells and tissues [27] (for reviews see [21, 28-30]). Furthermore, high RT titers have been found in the plasma of lymphoma and breast cancer patients, which drop dramatically after cancer treatment [31-32]. Recent studies independently indicate that cancer genomes are often “flooded” with hundreds of new, potentially mutagenic, retrotransposition events that can affect genome function and compromise its stability [10, 33-34], creating a mutant genomic environment favourable for tumor progression.

We previously contributed to pinpoint a role of LINE-1-encoded RT in cancer. We showed that RT inhibition, by either LINE-1-targeted RNA interference (RNAi) [35-36], or by RT inhibitory drugs [35, 37-38], drastically reduced cell proliferation and promoted differentiation in tumor cell lines and in *ex vivo* blasts from leukaemia patients (reviewed in [39]). We also found that the RT inhibitor efavirenz (EFV) [40] effectively antagonized the growth of human cancers xenografted in nude mice *in vivo* [35]. These data suggest that the endogenous RT might represent a new therapeutic target and RT inhibitors can be effectively used in oncology [41-42]. Indeed, phase II clinical trials using EFV are currently in progress to treat metastatic prostate carcinoma patients [43].

In contrast with the empirical demonstration of the therapeutical efficacy of RT inhibitors, the mechanistic implication of RT in tumor progression is still elusive. It has been noted that, in principle, RT-dependent retrotransposition events might either have a “driver” potential (i.e., induce genetic changes that promote cancer progression) or represent “passenger” mutations, not actively conferring growth advantage [44]. Our current knowledge of the retrotransposon molecular landscape during tumor growth is not sufficiently detailed to clarify this issue. In addition, the onset of retrotranposon activity during tumor growth remains so far unidentified.

Here we have investigated retrotransposons and LINE-1-encoded RT in the genesis and progression of breast cancer. We have used the transgenic mouse strain MMTV-PyVT, which expresses the polyomavirus middle T Antigen (PyVT) under the control of the mammary mouse tumour virus (MMTV) promoter, acting as an oncogene and causing the spontaneous growth of multifocal breast adenocarcinoma in 100% of females [45]. This strain provides a well-characterized, homogeneous and reproducible model for staging and following up breast carcinoma progression. We report that expression of LINE-1 and SINE B1 retrotransposons, at the level of both RNA and LINE-1-encoded proteins, bursts up in very early stages (stage 1) in breast tissue of transgenic animals and further increases in later stages of tumor progression (stages 4-6); in contrast, both retroelements are expressed at barely detectable levels in breast tissues of healthy controls. Consistent with the activation of retrotransposon expression, we have detected a significant amplification of both the LINE-1 and SINE B1 copy number, starting at stage 1 and continuing throughout tumor progression. These findings together support the conclusion that transcriptional deregulation and genomic variations of LINE-1 and SINE B1 copy numbers are distinctive features of a genomic landscape permissive for tumor onset and progression.

## RESULTS

### Expression of LINE-1-encoded protein product in tumor progression

As a follow up on the empirical evidence that LINE-1-encoded RT is implicated in tumorigenesis, it was of interest to assess LINE-1 retrotransposon expression during breast cancer progression. To this end, we undertook a systematic characterization of breast tumors withdrawn from MMTV-PyVT transgenic females at different times after birth, corresponding to progressively advanced cancer. Healthy breast tissues were obtained from females of the same strain (FVB/N) from which the MMTV-PyVT transgenics were generated.

The results of tissue histological analysis and immunohistochemistry of breast cancer markers are summarized in Table 1. Explanted breast tumor tissues were analyzed at sequential stages of progression (stages 1-6, as detailed in Table 2), demonstrating that murine cancer tissues progressively acquire the typical structural and histological features used for human breast cancer staging (examples in Figure S1): specifically, stage 1 tumors (panels B) exhibited histological grade 1 (low grade), extensively retaining the well-differentiated organization of non-pathological tissue (in panels A for comparison), yet showing areas with hyperproliferation of both ductal and lobular epithelia. At stages 2 (panels C) and 3 (panels D) the tumor tissue was still moderately differentiated, corresponding to histological grade 2 (or intermediate grade), with mixed In Situ Lobular Carcinoma (LCIS) and Ductal Hyperpalsia (DHy); at stage 3, in addition, intraductal necrotic material and rare infiltrating tumor cells could be appreciated, indicative of increased malignancy *versus* stage 2. In stages 4 (panels E), 5 (panels F) and 6 (panels G), the tumor tissues lost their differentiated morphology, becoming completely disorganized with an irregular pattern, corresponding to histological grade 3 (high grade); this was accompanied by histological features of increasing malignancy from stages 4 to 6, as detailed in Table 1. Widespread vascularization and vessel invasion became evident at stage 6, suggestive of progression toward the metastatic disease [46]. In parallel, we assessed the expression of proliferation marker Ki67, epidermal growth factor receptor (ERB2) and estrogen receptor (ER) by immunohistochemistry (Figure S2, summarized in Table 1). ERB2 and ER are routinely used in breast cancer diagnosis and exhibit opposite trends during cancer progression: ER is typically down-regulated, while ERB2 becomes overexpressed compared to earlier stages [47]. In assessing Ki67, ER and ERB2 markers, we considered both the frequency of positive cells and the staining intensity in the tissue samples. All three markers were consistently modulated in progressive tumor stages, indicating in particular: i) an increase in cell proliferation, with highest enrichment of Ki67-positive cells at stage 6 (50.9%, *versus* 9% in normal breast and 18.2% in stage 1); ii) decreasing ER expression, virtually disappearing at stage 5 (only rare scattered cells with weak signals are visible at stage 6), and iii) increased ERB2 expression, peaking at stage 5. Based on these histological and immunohistochemical features, we conclude that the progression defined in the murine breast cancer model faithfully recapitulates human breast cancer progression [47].

**Table 1:** Histological and immunohistochemistry analysis of the expression of tumor markers and LINE 1 ORF2p in sequential stages of cancer development

Samples	Histology		Ki67	Epidermal Growth Factor Receptor (ERB2)		Estrogen receptor (ER)		LINE 1-ORF2p	
	Histological grade	Histological features	Positive cells (%)	Positive cells (%)	Expression^a^	Positive cells (%)	Expression^a^	Positive nuclei (%)	Expression^a^
Normal Breast	Normal tissue	Normal breast	9%	>30% <50%	+	>50%	+±	Rare nuclei	±
Stage 1	Low	Lobular and Ductal Hyperplasia	18.2%	>10% <30%	+	>10% <30%	+±	<10%	+±
Stage 2	Intermediate	Mixed In Situ Lobular Carcinoma (LCIS) and Ductal Hyperpalsia (DHy)	22.9%	>30% <50%	++	>10% <30%	+	<10%	+
Stage 3	Intermediate	LCSI + DHy with intraductal necrotic material and rare infiltrating tumor cells	30.8%	>50%	++	>10% <30%	+	>30% <50%	++±
Stage 4	High	In Situ carcinoma with mixed lobular and ductal phenotype (LCSI predominant)	32.8%	>50%	++	>10% <30%	+	>10% <30%	++
Stage 5	High	Infiltrating Lobular and Ductal carcinoma	48.2%	>50%	+++	−	−	>30% <50%	+++
Stage 6	High with vessel invasion	Infiltrating Lobular and Ductal carcinoma with widespread vascularization and vessel invasion by tumor cells	50.9%	>30% <50%	++	<10%	±	>50%	++±

a Intensity vs the control background performed using only secondary but no primary antibody. Results show mean values from 3 animals

**Table 2:** Sources of breast tissues, tumor stages and age at which mice were sacrificed for sample withdrawal

Animal strains	Age
NB (healthy FVB/N animals)	Various ages between 30 and 105 day
Stage 1 (MMTV-PyVT)	30 days after birth
Stage 2	45 days after birth
Stage 3	60 days after birth
Stage 4	75 days after birth
Stage 5	90 days after birth
Stage 6	105 days after birth

**Figure 1:**
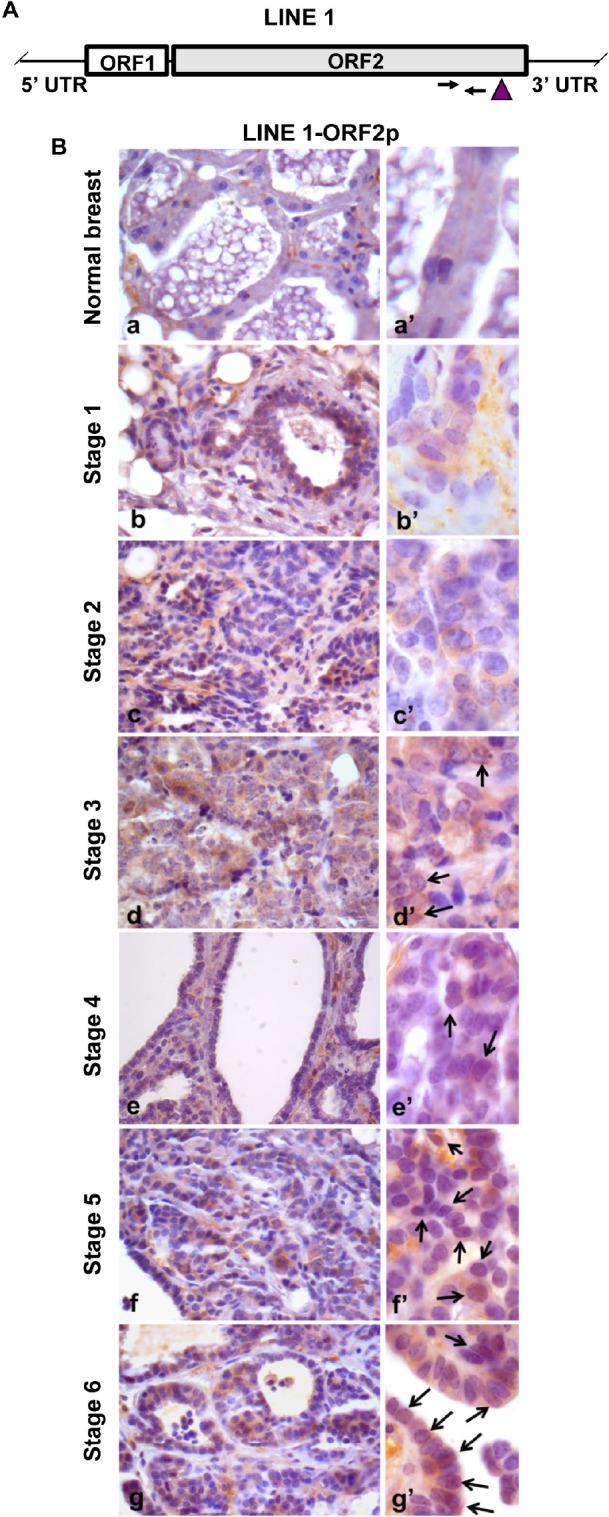
Immunohistochemical detection of ORF2p during murine mammary cancer progression A. Structure of a full-length LINE-1 (L1) element. Arrows mark the position of oligonucleotide pairs used for q-PCR and the vertical arrowhead identifies the protein domain recognized by ORF2p-specific antibody. B. Immunohistochemical analysis of LINE-1ORF2p in normal breast (a) and in tumor tissues explanted from transgenic mice at sequential stages of breast cancer development from stage 1 to 6 (b to g). High magnification panels (a' to g') depict the intracellular distribution of LINE-1 ORF2p. Arrows point to positive nuclei for LINE-1 ORF2p.

**Figure 2:**
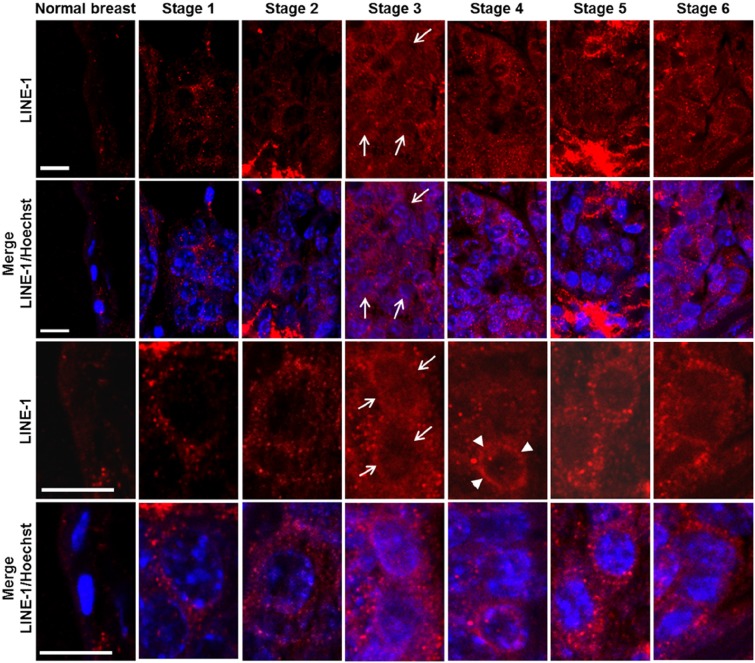
Confocal microscopy of ORF2p intracellular distribution during murine mammary cancer progression The top row panels depict ORF2p-specific IF, the bottom rows panels show merged images of LINE-1 ORF2p (red channel) and Hoechst-counterstained nuclei (blue channel). The arrows point to LINE-1 ORF2p positive nuclei and arrowheads to perinuclear accumulation of LINE-1 ORF2p. Panels in rows 3 and 4 show high magnification image details. Bars, 10 micrometers.

**Figure 3:**
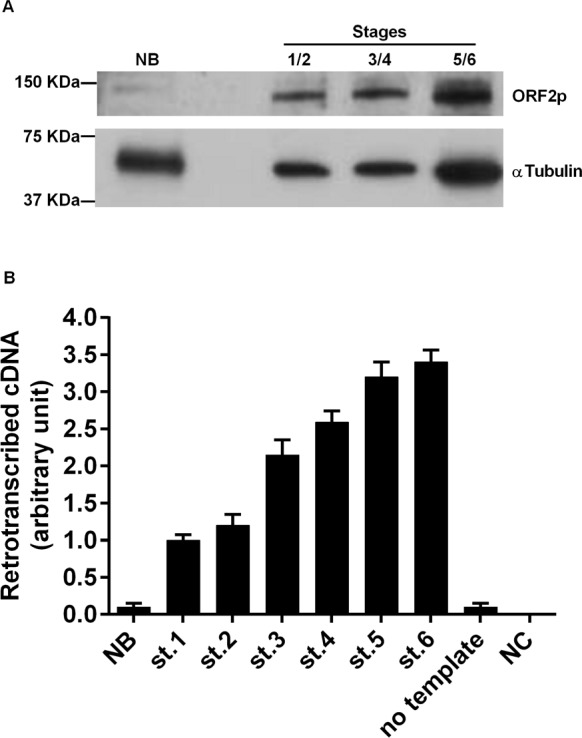
RT activity in murine breast cancer tissues A. Western blot analysis of ORF2p (upper panel) and alpha-tubulin (lower panel) in tissue extracts from normal breast (NB) and breast cancer (stages 1+2, 3+4, and 5+6 were pooled). B. RT activity functional assay after incubation of MS2 phage RNA with extracts from breast carcinoma tissues. Histograms represent the retrotranscribed cDNA yield from each reaction; means and SD values from three independent assays are expressed in arbitrary units.

We next investigated LINE-1 expression in relation to relevant parameters (i.e., histological grade, proliferation rate, ER and ERB2 levels) during breast cancer onset and progression. LINE-1 ORF2 (ORF2p) encodes a single polypeptide (145 kDa in molecular mass) that contains three highly conserved domains, corresponding to endonuclease (EN), reverse transcriptase (RT) and a cysteine-rich motif (CYS) [48], respectively. RT levels were assayed by immunohistochemistry using an antibody raised against the ORF2p C-terminus (Figure 1A). Virtually no ORF2p-specific signal was detected in normal breast tissue (Figure 1B, panel a, magnified in a'; data are quantified in Table 1), except for some occasional background, mainly on stromal spindle cells surrounding the alveoli and also present in no-primary antibody controls (Figure S2, panels A', B'). Weak signals were appreciated from stage 1 (Figure 1B, panels b and b'), with a predominant cytoplasmic localization, and increased through stages 2 (Figure 1B, panels c and c') and 3 (Figure 1 B, panels d and d'); the highest abundance, both in terms of signal intensity and of positive cell percentage, was observed at stages 5 (Figure 1B, panels f and f') and 6 (panels g and g'). We noticed that LINE-1 ORF2p signals were cytoplasmic in early stages but accumulated in nuclei during cancer progression (arrowed examples in high magnification images in Figure 1B, panels d' to g'). Confocal immunofluorescence microscopy was also employed to further investigate LINE-1-encoded ORF2p in breast cancer tissues, confirming a remarkable increase in ORF2p abundance during breast cancer progression (Figure 2); parallel to the increased abundance, we norticed cells exhibiting ORF2 perinuclear accumulation from stage 3 (arrowheads) and clear nuclear signals at stages 5 and 6. Western blot assays of total protein extract confirmed the antibody specificity (Figure 3A): the full-length 145 KDa ORF2 translation product was clearly detectable in tissue extracts from all tumor stages, but not in normal breast tissue extract (lane NB).

### Functional assays of LINE1-derived RT in cancer progression

We wondered whether breast tissues at different stages of cancer progression, in which LINE-1 ORF2p abundance gradually increased, were actually endowed with a parallel increase in their overall retrotranscriptional activity. To address that question we used a PCR-based assay (details in [37]), in which the RNA genome of the MS2 phage was used as a pure RNA template, and protein extract from breast tumor stages provide the source of RT activity to be tested. Identical amounts of protein extract from tumor tissues at all stages were loaded in the reactions. Measuring the yield of retrotranscribed cDNA copies in direct qPCR assays enabled us to assess the RT enzymatic activity. The results quantified in Figure 3B indicate that normal breast tissue extract (lane NB) harbor very low levels of functional RT activity. Retrotranscription was however markedly up-regulated in tumor tissue extract, starting from stages 1 and 2, and further increasing at later stages of progression.

### RNA overexpression of LINE-1 and SINE B1 retrotransposon families during tumor progression

The increased ORF2p abundance and retrotranscriptional activity detected during breast cancer progression prompted us to investigate whether LINE-1 transcription was also modulated in parallel. We also asked that question for SINE B1 retrotransposons, the RNA transcripts of which are not translated into proteins. RNA was extracted from tissues of progressively advanced breast cancer stages and amplified by qRT-PCR, using LINE-1 ORF2- or SINE B1-targeted pairs of oligonucleotides (details in Materials and Methods). RNA from normal breast (NB) tissue of healthy females was used for control.

**Figure 4:**
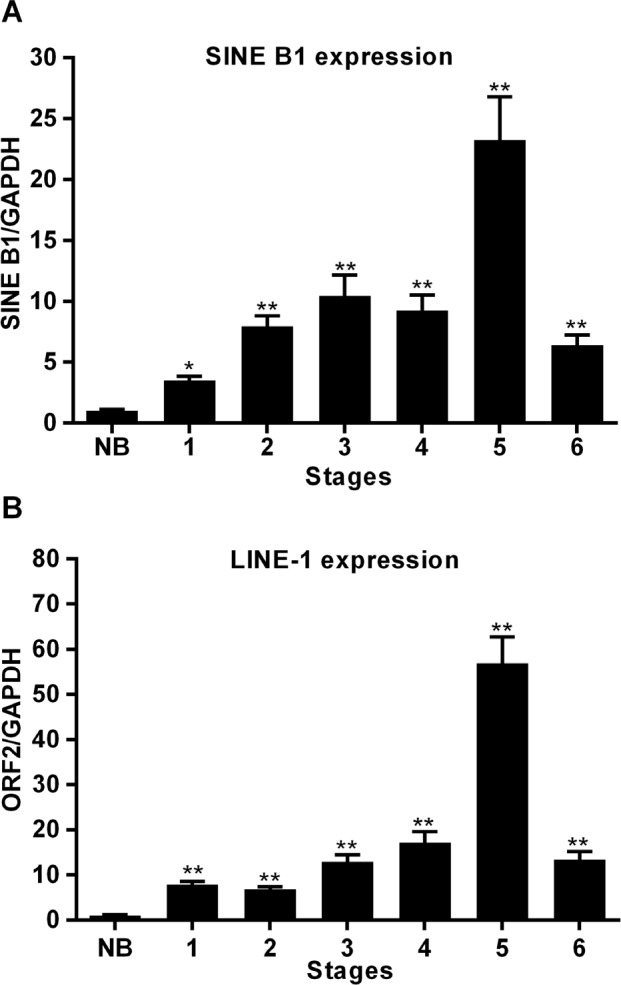
Transcription of SINE B1 and LINE-1 RNA in normal breast tissue (NB) and at the indicated stages of tumor of progression A. SINE B1 transcribed RNA. B. LINE-1 transcribed RNA. Transcription values were assessed by qRT-PCR and normalized to *gapdh*. Mean and SD values were calculated from three independent RT-PCR reactions, each in triplicate. ** Highly significant differences, * significant differences, compared to values from NB breast tissue.

As shown in Figure 4, RNA transcription from both SINE B1 (A) and LINE-1 (B) elements was dramatically up-regulated in tumor stages compared to healthy breast tissue, in which the basal level of RNA transcription was close to zero for both retrotransposon families. Taking *gapdh* as an internal control, transcriptional levels peaked at stage 5 for both SINE B1 and LINE-1 retroelements and, somewhat unexpectedly, decreased in stage 6, though remaining significantly higher than in healthy tissues. The timing of transcriptional up-regulation, parallel to the immunohistochemical detection of LINE-1-encoded ORF2p, precedes the appearance of extensive histological alterations typical of breast cancer progression. Thus, the RNA transcription yield of both retrotransposon families, though being mediated by different polymerases (Pol III for SINE B1, Pol II for LINE-1), is activated at early stages of tumor onset in a concerted manner.

### Amplification of LINE-1 and SINE B1 copy number during tumor progression

We finally assessed whether the increased abundance of SINE B1 and LINE-1 RNA transcripts in cancer cells provides additional templates for reverse transcription to generate new retrotransposon copies. DNA samples were extracted from staged breast tumor tissues as described for RNA transcript analysis and analyzed by direct qPCR to assess copy number variations using the *tfrc* single- copy gene as an internal normalization standard.

**Figure 5:**
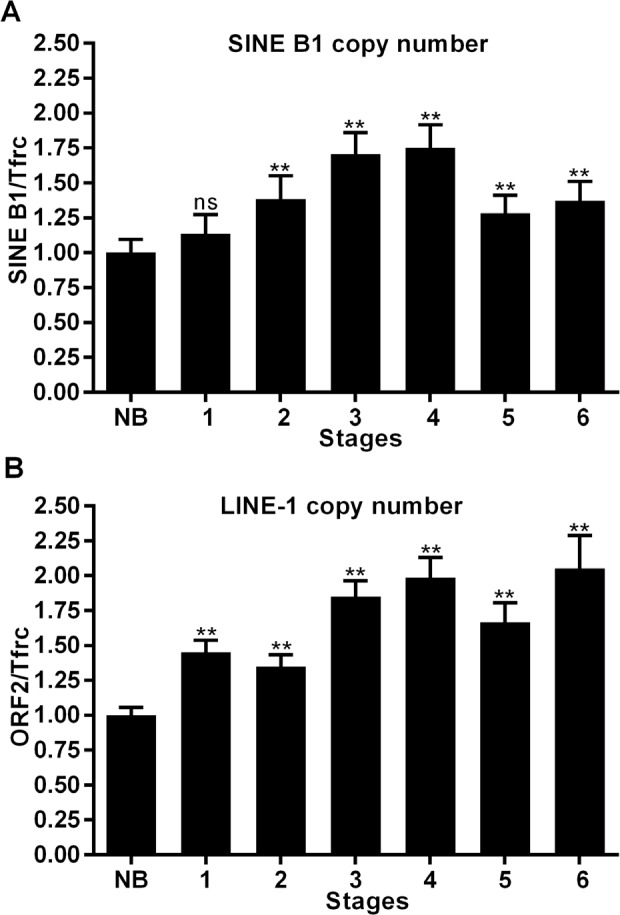
Copy number variations of SINE B1 and LINE-1 retroelements during breast cancer progression A. SINE B1 copy number variations. B. LINE-1 copy number variations in genomic DNA extracted from normal breast tissue (NB) and from the indicated tumor stages (1-6). Copy number values were assessed by q-PCR and normalized to the *tfrc* gene. ** Highly significant differences, * significant differences compared to values from NB breast tissue. NS, not significant.

Results in Figure 5 depict an amplification process involving both SINE B1 (A) and LINE-1 (B) copy numbers: the process is activated as early as stage 1 and progressively increases, peaking at stage 4 of tumor development. At that stage, a highly significant increase in copy numbers is observed for both elements relative to the level measured in normal breast tissue genomic DNA. The copy number of LINE-1 retroelements remained substantially unchanged in stages 5 and 6. The SINE B1 copy number showed some decrease in the same stages, yet remained significantly higher compared to that present in the genome of non-transformed cells. On the whole, these results suggest that a reverse transcription-mediated amplification process is triggered at cancer onset for both LINE-1 and SINE B1 retrotransposon families and continues throughout tumor progression.

## DISCUSSION

The present work builds upon our previous finding that the LINE-1-encoded RT activity plays a role in tumor onset and progression (see [39] for a review), as either LINE-1-specific RNAi [35-36], or drug-mediated [35, 37-38] RT inhibition, exert anti-cancer effects. Those studies were carried out using cancer-derived cell lines and cancer xenografts in murine models; they therefore could not pinpoint the timing at which the retrotransposon-encoded RT operates. This is a relevant question to gain deeper understanding of the role of RT in the genesis and progression of cancer. To address that question, here we have investigated for the first time the LINE-1-encoded RT at tumor onset and in progressing stages, in a systematic and comprehensive manner, in relation to the malignancy grade and to histological hallmarks.

The MMTV-PyVT breast cancer-prone transgenic mouse model [45] offers the opportunity to study tumorigenesis in well-defined stages of progression, from early onset to metastatic disease, in animals with identical genetic background. The results reported here show for the first time that events occurring at distinct levels of retrotransposon function are concomitantly up-regulated very early at cancer onset: LINE-1 and SINE B1 RNA transcript abundance was up-regulated (Figure 4); in addition, both LINE-1 and SINE B1 families of retrotransposons underwent copy number amplification (Figure 5). Both events were activated early in breast carcinoma, before the massive appearance of histological alterations and expression of tumor markers. These circumstances suggest that increased ORF2p abundance, and the ensuing increase in RT enzymatic activity (Figure 3), represent starting features of tumor-prone rather than overt tumorigenic tissues (Table 1). Together with the evidence recalled above that RT down-regulation blocks cancer progression [35–37], the data support the conclusion that the activation of the retrotransposon machinery is not a simple passive consequence of cell transformation or tumor growth, but rather acts in cancer-promoting processes. The events characterized here emerge as components of a feed-forward loop during breast cancer progression, in which the abundantly transcribed RT-encoding LINE-1 RNA is translated into protein and provides an increasing source of functional RT activity; the latter reverse-transcribes its own RNA (i.e. the transcript copies that encoded it), as well as the RNA transcribed from non-autonomous SINE elements, generating new LINE-1 and SINE copies during cancer progression. The eventual integration of the newly synthesized retroelement copies may contribute to increase chromosomal instability [49–51], a condition favoring tumor progression. This would be consistent with a growing body of data showing unscheduled activation of retrotransposon functions in a variety of human cancers. Emerging data indicate that a naturally occurring siRNA-based LINE-1 silencing mechanism [52] is active in normal cells, yet is defective or suppressed in tumors [53], leading to hypomethylation of LINE-1 promoters [21, 54-55] and uncontrolled retrotransposon activity. The loss of control of these mechanism makes tumor cells highly permissive to the deregulated expression of LINE-1 and other retrotransposon families [56,57], the activity of which is repressed under non-pathological conditions. These findings integrate to define what is currently viewed as a retrotransposition-prone cancer genomic landscape [10, 33-34, 44]. The early overexpression of LINE-1 and SINE RNA, their genomic copy number increase, and the accumulation of ORF2-encoded protein likely provide the molecular tools that lead to progressive remodeling of the retrotransposition-prone cancer genome. The finding that ORF2p accumulates in nuclei in advanced cancer stages (Figures 1, 2 and Table 1) is consistent with this picture, suggesting that ORF2 proteins, containing both RT and endonuclease activities [58], favor the integration of newly reverse-transcribed LINE-1 and SINE B1 copies in the host genome. Interestingly, LINE-1-encoded ORF1 and ORF2 proteins were reported to have a predominant nuclear localization in human breast tumors with poor prognosis, lymph node metastasis and the worst patient survival compared with patients with cytoplasmic expression [59, 60].

It is important to note, however, that the amplification of LINE-1 and SINE B1 copy number in cancer compared to normal breast tissue (Figure 5) does not necessarily imply a parallel increase in the rate of actual integration events: a proportion of newly synthesized retroelement copies may remain extrachromosomal and exert epigenetic effects in trans. That possibility might underlie the observation that the genomic copy number, at least for SINE B1 elements, does not continue to increase throughout terminal cancer, but peaks at stage 4, preceding the most dramatic stages of phenotypic transformation.

In conclusion, the present data support the view that a retrotransposon-based mechanism is activated early at tumor onset and remains active throughout the subsequent stages of tumor growth, with a progressive mechanism of retrotransposition expansion. The finding that the retrotransposon machinery is activated early in tumorigenesis substantiates the rationale for regarding the LINE-1-encoded RT protein as a novel early tumor marker of clinical relevance, with potential diagnostic value [57].

## METHODS

### Ethic statement

Investigation using animals has been conducted in accordance with the ethical standards and according to the Italian DL 116/92, enforcing the European Directive 86/609/EEC on Laboratory Animal Welfare. and has been approved by the authors' institutional review board.

### Mouse strains

MMTV-PyVT transgenic mice (generated from mouse strain FVB/N) [45] were purchased from the Jackson Laboratory (Bar Harbor, MI, USA). Transgenic mice were sacrificed at different times after birth (see Table 2); mammary tissues were dissected and stored in liquid nitrogen.

### Genomic DNA extraction, RNA extraction and cDNA synthesis

Genomic DNA was extracted from mouse breast tissues by standard methods; briefly: tissues were lysed in lysis buffer (50 mM Tris, 10 mM EDTA, 1% SDS, 50 micrograms/ml proteinase K) overnight at 37°C and genomic DNA was purified through phenol/chloroform extractions, extensively treated with RNase A (Sigma-Aldrich), ethanol precipitated and resuspended in sterile water. DNA samples were quantified using NanoDrop 1000 (Thermoscientific, Wilmington, DE). Total RNA was isolated from mouse tissues using the Total RNA Mini Kit (GeneAid) following manufacturer's instructions, with the exception that an additional DNase I Amp Grade (Invitrogen) step was included. 1 microgram-aliquots of purified RNA were incubated with 50 ng random hexamer primers in cDNA synthesis reactions using the ThermoScript RT-PCR System (Invitrogen).

### RNA expression and copy number evaluation

Quantitative real time PCR (qPCR) was performed in a 7500 Fast Real-Time PCR System (Applied Biosystems) under the following conditions: one cycle of 50°C for 2 min, one cycle of 95°C for 10 min, 40 cycles of 95°C for 15 s, 60°C for 1 min. The results were analyzed with qPCR 7500 Software Download v. 2.0.6.

LINE 1 ORF2-specific primers and probes were reported previously [61]. SINE B1-specific primers and probes were designed using the Primer Express software V3.0 based on the consensus sequence [62]: SINE B1 Forward: 5'-TGG CGC ACG CCT TTA ATC-3'; SINE B1 Reverse: 3'-TGG CCT CGA ACT CAG AATCC-3'; SINE-B1 Probe 6FAM- ACT CGG GAG GCA GAG G- MGB. Five cDNA serial dilutions were used to assess optimal conditions for SINE B1 amplification efficiency: the primer efficiency was verified by linear regression to the standard curve with a slope near −3.30. The murine single-copy genes *tfrc* and *gapdh* (both from Applied Biosystems) were used for copy number evaluation and RNA expression, respectively. TaqMan-MGB probes were also from Applied Biosystems.

LINE-1 and SINE B1 content were determined by the ΔΔCT method and plots represent relative quantity (RQ) of amplification compared to normal breast (NB), which was taken as 1. Samples from three independent experiments were analyzed by qPCR and each sample was routinely analyzed in triplicate. Homogeneity of each amplicon product was confirmed by gel electrophoresis. All data obtained for ORF2 and SINE B1 copy numbers and expression across development were statistically analyzed in Multiple Comparisons versus Control Group (Bonferroni t-test). Statistically significant differences were evaluated using the one-way ANOVA test with Bonferroni correction.

### Total protein extraction and Western blotting

Total proteins were extracted from breast tissues by homogenizing 100 mg of tissue in a glass-teflon homogenizer in 1 ml RIPA buffer (50 mM Tris-HCl pH 7.5, 150 mM NaCl, 1 mM EGTA, 1mM EDTA, 1% Igepal CA-630, 0.25 % sodium deoxycholate), supplemented with 1x complete mini EDTA-free protease inhibitors (Roche) and incubated for 30 min on ice. Extracts were then centrifuged at 13,000 × g (20 min, 4°C) and the supernatants were stored at −80°C. Protein concentrations in cell extracts were determined using the Coomassie Plus Protein assay Reagent (Pierce, Rockford). 50 micrograms of total proteins were diluted in 50 microliters Laemmli buffer (31.5 mM Tris-HCl, pH 6.8, 10% glycerol, 1% SDS, 100 mM DTT, 0.005% Bromophenol Blue), heated at 90°C for 10 min and fractionated through 7.5% Mini-Protean TGX Precast Gel (Bio-Rad) at 150 V. Fractionated proteins were electrophoretically transferred with Trans-Blot Turbo Transfer System (Bio-Rad) on a nitrocellulose membrane that was then blocked in TBS-T (50 mM Tris-HCl, pH 7.6, 150 mM NaCl, containing 5% milk and 0.1% Tween-20) for 1h at room temperature. The filter was incubated with rabbit polyclonal antibody to LINE-1 ORF2p (1:400 dilution, Santa Cruz Biotechnology) overnight at 4°C, washed in TBS-T and further incubated with goat anti-rabbit IgG (H+L)-HRP conjugated secondary antibody (Bio-Rad, 1:10,000 dilution) for 1 hour. Signals were revealed using the enhanced chemiluminescence system (Clarity Western ECL Substrate, Bio-Rad). Routinely, the membranes were stripped and reprobed with anti-alpha-tubulin antibody (1:20,000 dilution, Sigma-Aldrich).

### RT enzymatic activity assay

RT activity was evaluated as described [37] with minor modifications. Briefly, 20 ng of DNAse I Amplification Grade (Invitrogen) pre-treated MS2 phage RNA (Roche Diagnostics) were used as RNA template after pre-incubation with 400 nM of MS2 reverse primer (see below) at room temperature for 30 min. cDNA synthesis was carried out using the Thermoscript RT–PCR system, replacing commercial RT with 6 micrograms of total protein extract (see below) from tissues. Reaction mixtures were incubated at room temperature for 1 h followed by 5 min at 85°C. 1 microliter of RNase H was added to each sample and further incubated at 37°C for 20 min. Control reactions were set up by either omitting cell extract, or omitting template, or adding 1 microliter of ThermoScript RT enzyme (positive control). 2 microliters from each reaction were amplified with IQ5 Real Time PCR (Bio-Rad), using SsoAdvanced SYBR Green Supermix (Bio-Rad) and 400 nM of MS2 forward (5'-GGAGCCTGATATGAATATGTACC) and reverse (5'-GATAAGTCTATCGTCGCAAGC) primers. Each reaction was repeated three times in triplicate.

### Histological and immunohistochemical analyses

Normal and tumor breast tissues explanted from transgenic mice were routinely fixed in 10% buffered formalin and embedded in paraffin. Sections from each paraffin block were sliced and stained with hematoxylin-eosin for histological examination. For immunohistochemical staining, sections were collected on APES-coated slides (Dako) and examined for the expression of Ki67, ER, ERB2 and LINE-1 using the following antibodies: rabbit polyclonal anti-LINE1 antibody (1:100 dilution, Santa Cruz Biotechnology); rabbit polyclonal anti-ErbB2 antibody (1:350 dilution, Abcam); mouse monoclonal anti-ER antibody (1:100 dilution, Santa Cruz Biotechnology); rabbit monoclonal anti-Ki67 antibody (1:100 dilution, Abcam). Tissue sections were incubated in 1% BSA for 15 min at room temperature, then overnight with specific primary antibody at the indicated working dilutions. Primary antibody was revealed by the streptavidin-biotin complex method using the KIT DAKO Cytomation LSAB 2® System HRP (Liquid DAB) and, after peroxidase reaction, sections were counterstained with hematoxylin. For each examined tumor specimen, background controls were performed on a section close to that used for immunostaining by omitting primary antibody. Quantitative analyses of proliferation rate (positivity for the proliferation marker Ki67) and of ER, ERB2 and LINE-1 ORF2p signals, were performed on three animals per group by evaluating the percentage of positive cells and the staining intensity (see criteria in Table S1).

### Confocal laser scanning microscopy (CLSM)

CLSM analyses of LINE-1 ORF2p were carried out on paraffin- embedded tissues by indirect immunofluorescence using rabbit polyclonal antibody to LINE-1 ORF2p (1:100 dilution, Santa Cruz Biotechnology). Primary antibody was detected using Alexa Fluor 647-conjugated anti-rabbit IgG (Molecular Probes). Sections were counterstained with Hoechst (1:4,000 dilution). Samples were observed under a LEICA TCS SP5 confocal laser scanning microscope (Leica Instruments).

## Supplementary Tables and Figures




