# Supplementary material for: Increased expression and copy number amplification of LINE-1 and SINE B1 retrotransposable elements in murine mammary carcinoma progression
Source: Oncotarget. 2013 Aug 9;4(11):1882–93. doi: 10.18632/oncotarget.1188 (PMC3875756; doi:10.18632/oncotarget.1188)
Supplement: Supplementary file 1 [file oncotarget-04-1882-s001.pdf]

Increased expression and copy number amplification of LINE-1 and SINE B1 retrotransposable elements in murine mammary carcinoma progression – Gualtieri et al

Table S1. Quantitative criteria used in immunohistochemical and histological analyses.

Biomarker expression			Histology	
**Signal intensity (sample vs. control treated with 2ary antibody)**		**Cell positivity (%)**	**Severity of lesions**	**Histological grade**
– (same as background)	1	Absent	Normal	
± (moderately higher than background)	5	≤ 10%	Low	Grade 1
+ (higher than background)	10	>10% <30%	Intermediate no infiltrating cells	Grade 2
			Intermediate with infiltrating cells	Grade 2
++ (much higher than background)	20	>30% <50%	High mixed LCSI and DCSI	Grade 3
			High infiltrating LC and DC	Grade 3
+++ (dramatically higher than background)	30	≥50%	High infiltrating LC and DC with vessel invasion	Grade 3
