# Supplementary material for: Increased expression and copy number amplification of LINE-1 and SINE B1 retrotransposable elements in murine mammary carcinoma progression
Source: Oncotarget. 2013 Aug 9;4(11):1882–93. doi: 10.18632/oncotarget.1188 (PMC3875756; doi:10.18632/oncotarget.1188)
Supplement: Supplementary file 2 [file oncotarget-04-1882-s002.pdf]

Increased expression and copy number amplification of LINE-1 and SINE B1 retrotransposable elements in murine mammary carcinoma progression - Gualtieri et al

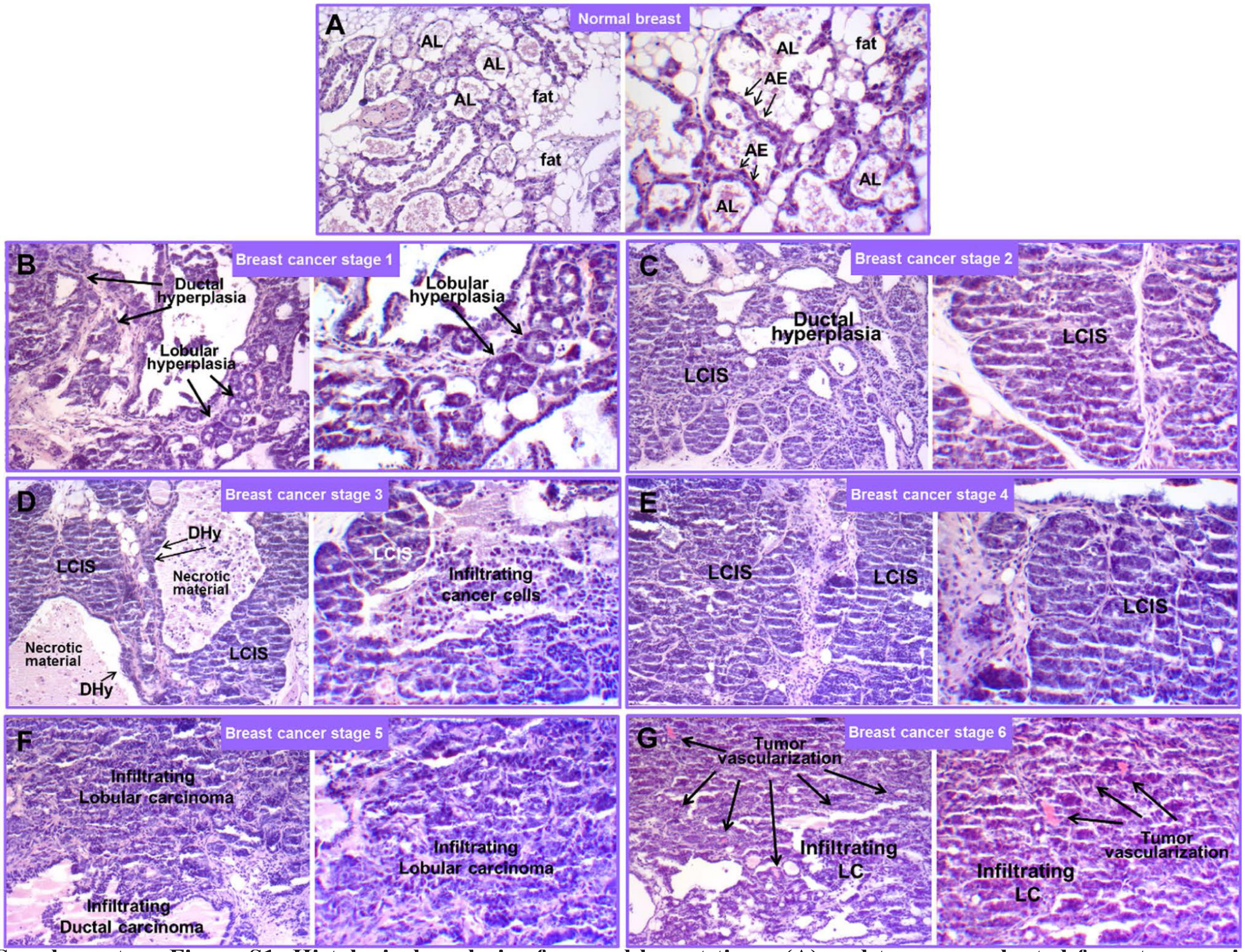

Supplementary Figure S1: Histological analysis of normal breast tissue (A) and tumors explanted from transgenic mice at the sequential stages of breast cancer development (B-G). In each panel, two representative fields at different original magnifications are shown (left 10x, right 20x). A) Normal breast; AL: Alveolar Lumen, AE: Alveolar Epithelium. B) Stage 1: large areas with hyperproliferation of both ductal and lobular epithelia are indicated by the arrows. C, D) Stages 2 and 3: in situ Lobular Carcinoma (LCIS) and Ductal Hyperpalsia (DHy) are mixed; at stage 3, intraductal necrotic material and rare infiltrating tumor cells are indicated. E) Stage 4: in situ carcinoma with mixed lobular and ductal phenotype; LCSI areas are predominant with respect to those of in situ Ductal Carcinoma (DCIS). F) Stages 5: infiltrating Lobular and Ductal carcinoma occupied the majority of tumor tissue. G) Stage 6: large areas with infiltrating Lobular Carcinoma (LC) and widespread vascularization are shown.

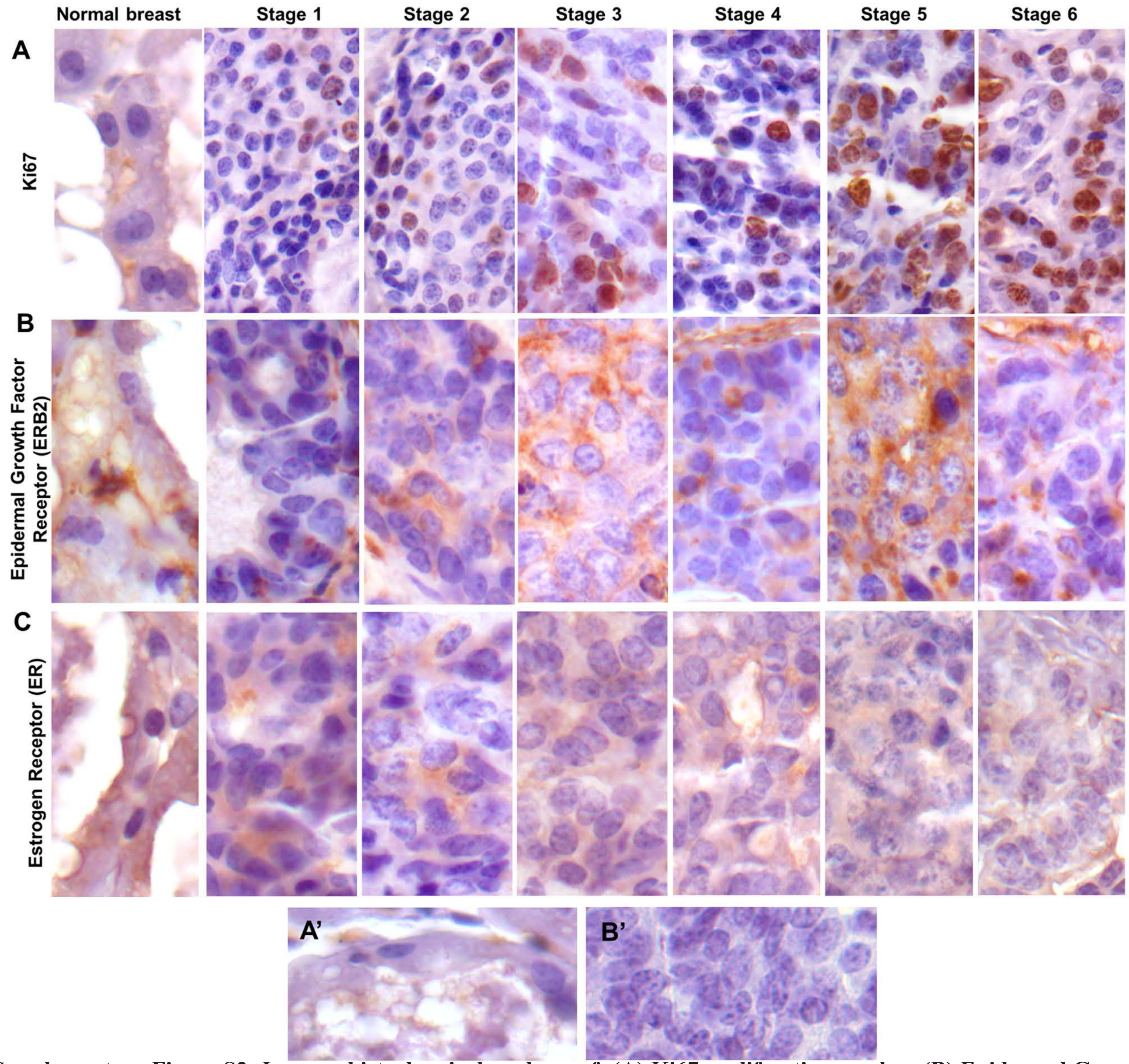

Supplementary Figure S2: Immunohistochemical analyses of: (A) Ki67 proliferation marker, (B) Epidermal Growth Factor Receptor (ERB2) and (C) Estrogen Receptor (ER) expression in normal breast and in tumors explanted from transgenic mice at the sequential stages of breast cancer development (from stage 1 to stage 6). Background controls performed by omitting the specific primary antibody on tissue sections from normal breast and stage 4breast cancer are shown in panels A' and B', respectively.
